# A quasi-experimental examination of weight-reducing dehydration practices in collegiate male rowers

**DOI:** 10.1186/s13102-021-00344-7

**Published:** 2021-09-25

**Authors:** Dayton J. Kelly, Sarah L. West, Nathan O’Keeffe, Liana E. Brown

**Affiliations:** 1 Department of Biology, Trent University, Peterborough, Canada; 2 Department of Psychology, Trent University, 1600 West Bank Drive, Peterborough, ON K9L 0G2 Canada; 3 Trent University, Peterborough, Canada; 4 Trent/Fleming School of Nursing, Trent University, Peterborough, Canada; 5 Northern Ontario School of Medicine, Sudbury, Canada

**Keywords:** Aviron, Fluid abstinence, Thermal exposure

## Abstract

**Background:**

Lightweight rowers commonly utilize weight loss techniques over 24-h before competition to achieve the qualifying weight for racing. The objective was to investigate, using a quasi-experimental design, whether changes in weight resulting from dehydration practices are related to changes in proxies of bodily systems involved in rowing and whether these relationships depend on the dehydration technique used.

**Methods:**

Twelve elite male rowers performed a power test, an incremental VO_2_max test, and a visuomotor battery following: weight loss via thermal exposure, weight loss via fluid abstinence and then thermal exposure, and no weight loss. The total percent body mass change (%BMC), %BMC attributable to thermal exposure, and %BMC attributable to fluid abstinence were used to predict performance variables.

**Results:**

Fluid abstinence but not thermal exposure was related to a lower total wattage produced on a incremental VO_2_max test (*b* = 4261.51 W/1%BMC, 95%CI = 1502.68–7020.34), lower wattages required to elicit 2 mmol/L (*b* = 27.84 W/1%BMC, 95%CI = 14.69–40.99) and 4 mmol/L blood lactate (*b* = 20.45 W/1%BMC, 95%CI = 8.91–31.99), and slower movement time on a visuomotor task (*b* = -38.06 ms/1%BMC, 95%CI = -62.09–-14.03).

**Conclusions:**

Dehydration related weight changes are associated with reductions in some proxies of bodily systems involved in rowing but depend on the dehydration technique used.

**Supplementary Information:**

The online version contains supplementary material available at 10.1186/s13102-021-00344-7.

## Background

Lightweight is a division of rowing in which athletes are required to weigh below a certain criterion 2 h prior to competition to be eligible for racing [1]. As greater height and lean mass is related to superior performance [2], it is common for athletes to weigh above the criterion and utilize weight loss techniques over the 24 h before competition to qualify for racing [1]. Dehydration is a common technique used to achieve weight loss because it allows for temporary reductions in weight without muscle protein loss, and hydration status can be at least partially restored over the 2-h rehydration window between weigh-ins and racing [3]. However, dehydration may still reduce performance [4].

Rowing performance relies on multiple components including an athlete’s efficient use of the aerobic and anaerobic energy systems, and capacity to produce power [5]. Additionally, the neuromotor system controls stroke execution and balance maintenance by continuously adapting the execution signal in response to sensory feedback [6, 7]. These systems have been demonstrated to be vulnerable to weight reduction via dehydration in settings outside of rowing [8]. It is unclear how and if changes in these systems contribute to changes in rowing performance following weight-reducing dehydration practices.

Some research suggests that the effect of weight-reducing dehydration practices may depend on the dehydration technique used [8, 9]. Four common options exist for rowers to achieve weight loss through dehydration before weighing in: diuretics, exercise, thermal exposure, and fluid abstinence [10]. As use of diuretics is illegal in elite sport [11] and exercise-induced dehydration appears to impair aerobic [12], anaerobic [9], and balance performance [13] to a greater extent than other dehydration techniques, these are poor candidates for rowers. Instead, rowers may choose to reduce weight by using either thermal exposure or fluid abstinence. Previous work [3, 4, 14–17] has demonstrated effects of large amounts of dehydration on 2000 m rowing performance: a common performance test in rowing. Within a pilot study [18], we found that this performance test may be negatively affected by smaller amounts of dehydration but that this effect depended on the technique used to elicit dehydration. Currently, there is a need to better understand how smaller amounts of dehydration effect rowing performance, the physiologic systems involved, and whether the dehydration technique used matters.

The present study examined elite collegiate rowers within an exploratory, quasi-experimental, within-participants design to investigate whether changes in weight resulting from mild weight-reducing dehydration practices are related to changes in bodily systems involved in rowing. Further, we sought to determine whether these relationships were dependent on the technique through which weight loss was achieved. We examined proxies of the aerobic energy system, anaerobic lactic energy system, anaerobic alactic energy system, and the neuromotor control system following weight loss via thermal exposure and fluid abstinence. Affected systems may contribute to poorer rowing performance following weight-reducing practices.

## Methods

### Participants

A total of 6 heavyweight and 6 lightweight competitive male rowers participated in the study (Table 1). Females were not included because body weight and total body water vary idiosyncratically with the menstrual cycle [19]. Participation was restricted to individuals ≥ 16 years who had competed at least 1 season at the university level or were members of a Canadian National Rowing Hub. All methods, including the study experiments and dehydration protocols (developed in consultation with coaches and athletes), were approved by the Trent University Research Ethics Board (REB#25493) and complied with the Declaration of Helsinki. All participants provided informed written consent prior to participating in the study.

**Table 1:** Demographic characteristics of the sample separated by weight class

	HWT (n = 6)		LWT (n = 6)	
	*M*	*SD*	*M*	*SD*
Age (yrs)	22.17	5.81	19.50	2.22
Weight (kg)	91.52	5.34	74.73	2.87
Height (cm)	189.33	4.61	183.67	4.71
VO_2_max/kg (mL/kg/min)	52.75	3.71	58.75	4.20

HWT = Heavyweight rowers, LWT = Lightweight rowers

### Design

Rowers completed an incremental VO_2_max test, peak power test, and 3 visuomotor tasks after 3 different weight loss conditions: once following weight loss through thermal exposure (SAU), once following weight loss through fluid abstinence and thermal exposure (FA + SAU), and once following no weight loss (CON). These hydration manipulations were used to achieve varying amounts of weight loss that further varied in the amount of each dehydration technique used to cause it. We quantified the observed amount of weight loss owed to each dehydration technique by measuring the change in weight across the timespan allotted for it on each of the 3 testing days. We summed these values to get the total weight change on each day. Through this process, we obtained a weight loss for each participant on each testing day associated with fluid abstinence, thermal exposure, and their sum (which approached zero on CON day because they were unexposed during the timespans). These changes in weight were used as the independent variables in our analyses (further details follow). Participants were tested on 3 separate days within a 2-week period and experienced the weight-loss conditions in a counterbalanced order. Blood osmolality, urine osmolality, and 2 dehydration questionnaires were recorded with body mass to assess whether this weight loss could be attributed to dehydration.

### Procedure

Baseline body weight, from which the weight reduction was calculated, was determined from a series of 5 baseline weigh-ins that were completed on non-test days immediately prior, during, and immediately following the study period. On the first testing day involving weight loss, participants attempted to achieve a weight reduction of 2.5% their average body mass by an official weigh-in time. This target weight loss was used as a guide to enable participants to reasonably match weight loss on future testing days. This target weight loss was selected based on findings from a pilot study [18]. On a participant’s second testing day involving weight loss, they attempted to match their previous weight loss. In all conditions, participants were required to fast from midnight until after the official weigh-in to minimize the impact of differing food consumption on body weight.

On the SAU day, the participants achieved weight loss by sitting in a sauna. On the FA + SAU day, participants were instructed to abstain from fluid intake for 15 h over the evening and night prior testing to achieve weight loss. Similar fluid restriction protocols have been previously employed by other authors [16, 17, 20, 21]. This fluid restriction protocol has been previously used over 12 h within a pilot study to induce 1–2% body mass loss [18]. This was followed by time in the sauna, if necessary. In both conditions, exposure to the sauna (70 °C) was provided in 15-min increments up to a total of 60 min. Temperatures within this range have been previously used to elicit dehydration [22, 23] and were used within our pilot study [18]. If at the end of any of these 15-min increments the target weight loss was achieved, the participant was exempt from further time in the sauna and maintained their weight loss until the official weigh-in time. Sauna exposure on FA + SAU day was included to enable matched weight loss across testing days and to ensure that total weight loss was not correlated with dehydration technique. On the CON day, the participants’ weight was not manipulated. Participants waited in the laboratory until their official weigh-in time for a matched amount of time (1 h and 15 min); their hydration was not restricted during this time.

Mimicking international competition, participants were provided 2 h to rehydrate between their official weigh-in time and exercise testing. It was recommended they rehydrate aggressively. Water, sport electrolyte drinks and breakfast foods were provided, and participants recorded the fluid and food they consumed. They completed a 24-h diet recall for the day prior (50 min following their official weigh-in) and a battery of visuomotor tests (80 min following their official weigh-in). This 24-h diet recall was also completed the day prior to testing to assess fluid and food intake 2 days prior testing.

Participants then completed a 10-min warm up period at a self-prescribed intensity, a peak power test, and, finally, an incremental VO_2_max test. All tests were completed within 90 min of the end of the rehydration window. All protocols were completed between 7 am and 1 pm under thermoneutral conditions.

To track weight loss/dehydration over the testing day(s), participants were weighed and completed thirst and dehydration symptomology questionnaires upon arrival, at the time of the official weigh-in and following the rehydration window. Blood and urine samples were taken at the time of the official weigh-in and following the rehydration window.

### Instruments and tasks

#### Body mass change

All body mass measurements were completed post-voided on a scale in pre-weighed clothes that were factored in (Health-O-Meter 349KLX, Health-O-Meter Professional, McCook, USA). Baseline body mass measurements were completed in the morning (6AM-9AM) after fasting from midnight onwards (without restricting water consumption). These baseline body mass measurements were completed on non-test days (CON, SAU, FA + SAU). Weight changes were expressed as percent changes in body mass (%BMC).

The total change in weight (total%BMC), the change in weight due to thermal exposure (thermal%BMC), and the change in weight due to fluid abstinence (abstinence%BMC) was calculated for each testing day (CON, SAU and FA + SAU; see equations in Additional file 1). Each measure should approach 0 on CON day. Weight changes due to fluid abstinence and thermal exposure were considered the observed weight changes over the time allotted for each (i.e. abstinence%BMC = the weight change at arrival; thermal%BMC = the weight change between arrival and the official weigh-in). The change in weight over the rehydration window was also calculated. The equations for determining total%BMC and partitioning abstinence%BMC and thermal%BMC are provided in the supplementary material.

#### Additional measures of hydration

We checked our hydration manipulation by assessing the athlete’s blood plasma osmolality (mmol/L) and urine osmolality (mmol/L). Blood was collected in BD PST Gel and Lithium Heparin vacutainers according via standard phlebotomy procedures [24] and immediately centrifuged (relative centrifugal force: 1000; 10 min). Urine samples were self-collected mid-stream. Blood and urine samples were aliquoted and subjected, in triplicate, to freezing point depression osmometry (3320 Single-Sample Micro Osmometer, Advanced Instruments, Norwood, USA). The average osmolality was recorded for each specimen.

Additionally, questionnaires were used to measure rowers’ perception of thirst (Visual Analogue Scale of Thirst; VAST) [25] and dehydration symptomology (Symptom Evaluation Subscale [SES] of the Sport Concussion Assessment Test 3; SCAT3) [26] and assessed relative to scores recorded during the baseline weigh-in sessions. The SCAT3 has previously been used to assess the effects of dehydration due to its overlap in symptomology with concussion [27].

#### Visuomotor tasks

Three tests were used to assess aspects of visuomotor control: the double-step, stop-signal, and interception tasks [28]. The tests were coded in Matlab (The Mathworks, Inc., Natick, USA), using the Psychophysics Toolbox extensions [29–31] and were presented on the detachable touchscreen of a personal computer (Surface Book 2, Microsoft, Redmond, WA). The code is available: https://github.com/lianabro/TabletTasks4Matlab. In brief, the double-step task assesses the participants’ ability to adjust ongoing movements to an unexpected change in target location. Targets jumped unpredictably (on 25% of trials) either forward or backward along the movement path. Movement time (ms) and end-point accuracy (mm) to the final target location were recorded. The stop-signal task assesses the ability to interrupt a planned and signaled movement in response to a variably-delayed signal to halt the movement (50 ms, 100 ms, 150 ms, 200 ms). The proportion of trials in which the participant was able to successfully interrupt their response in the most challenging delay condition (200 ms) was analyzed. The interception task assesses the ability to detect, predict, and use visual motion information to precisely intercept a moving target that varied in speed (13.5 cm/s, 21.4 cm/s, and 26.5 cm/s) and acceleration (0 cm/s [2] and 0.18 cm/s [2]). The percentage of successful hits and interception position and timing (ms) were analyzed for the most challenging speed (26.5 cm/s) and acceleration (0.18 cm/s [2]) condition. All testing was performed in a quiet, well lit room. The order of the visuomotor tasks was counterbalanced across participants but consistent over the testing days.

#### Rowing ergometer performance

Participants completed a maximal peak power test and an incremental VO_2_max test on a rowing ergometer (Concept II model D, Morrisville, USA). The same ergometer was used for all testing. Verbal encouragement was provided.

During the peak power rowing test, rowers completed two 10-stroke efforts on the rowing ergometer with the drag factor set to 200 (≤ 40 strokes/minute). The 10-stroke effort consisted of 6 preparatory strokes followed by 4 maximal strokes. Rowers were provided a 30 s rest between efforts. The highest wattage was recorded and averaged across the 2 tests. This test is a modified version of similar 5–15 stroke power tests that have been previously employed [32, 33]. This version of test was familiar to our athlete participants.

During the incremental VO_2_max test, rowers completed a stepwise-then-ramp incremental VO_2_max test to exhaustion with intermittent blood lactate sampling using a portable lactate meter (The Edge Lactate Analyser, ApexBio, Hsinchu, China). Expired gases were measured breath-by-breath using a metabolic cart (Cortex Metalyzer 3B, Cortex Medical, Walter-Köhn-Str. 2d, Leipzig, Germany). Wattage production and stroke rate were measured stroke-by-stroke using PainSled software (PainSled Indoor Rowing Dashboard [Version 0.7], Exerscreen).

The test began with a 1-min familiarization period at a wattage (W) equal to the first stage of the test. This starting wattage was calculated using a procedure that was adapted [34] and modified to calculate a wattage instead of split-time: 32 s was added to the rower’s self-estimated, current 2000 m test split-time; this split-time was converted to a wattage; and 35 W was subtracted from this number. The immediately proceeding stepwise portion of the test consisted of 3-min stages, separated by a 1-min rests, that increased by 35 W each stage. The ramp portion of the test began on the stage following the rest period at which blood lactate exceeded 4 mmol/L. A final a 1-min break was provided, and ramp portion began at 35 W higher than the last completed stage. During this portion, the wattage increased by 35 W every minute without breaks. A final 15 s maximal sprint was conducted when 3 consecutive strokes could not be completed at the prescribed wattage. Blood lactate was sampled within the first 30 s of each rest period and immediately following the final sprint.

Rowers were instructed to keep their stroke rate consistent across tests for each stage. The ergometer drag factor was set to the level at which the athlete trained most commonly and was consistent across tests. Data from the test was used to calculate the total wattage produced during the test, maximal and submaximal oxygen consumption/stroke efficiency (VO_2_max, VO_2_ at 2 mmol/L blood lactate, and VO_2_ at 4 mmol/L blood lactate), lactate production (wattage at 2 mmol/L blood lactate, wattage at 4 mmol/L blood lactate, blood lactate at test completion [maximum blood lactate], blood lactate prior test initiation [resting blood lactate]). Details regarding the calculation of these variables follow in the “Results” section.

#### Dietary assessment

An online 24-h diet recall tool (Automated Self-Administered 24-Hour-Canada [ASA24-Canada] Dietary Assessment Tool 2016; https://epi.grants.cancer.gov/asa24/) was used to determine total calorie, carbohydrate, fat, protein, sodium, and water intake for the 2 days prior testing. Food and fluid consumption recorded during the rehydration period was separately translated into these nutrient categories using the nutrient label on each food and fluid item.

### Statistical analysis

For each performance outcome variable we sought to determine: 1) whether the dehydration-related performance change was better explained by total%BMC or by the %BMC achieved via each technique (thermal%BMC and abstinence%BMC; i.e. does dehydration technique matter?), and 2) the magnitude of the dehydration-related performance change [change in the outcome measure for a 1% change in BMC (1%BMC)].

To do this, 2 linear mixed-effects models were used to assess the association between the weight change measures and each outcome variable (using participant ID as a random intercept term). In the present study, this model can be thought of as a within-subjects regression model from which the model weights (b) describe the change in the outcome measure for a 1% change in BMC. The first model contained only total%BMC and the second model contained thermal%BMC, abstinence%BMC, and their interaction. We compared the models using a likelihood ratio test. When the likelihood ratio test was significant, suggesting that dehydration technique affected the outcome, we interpreted only the second model. Otherwise, the first model was interpreted (the interpreted model is bolded, when significant, in all Tables; the uninterpreted model is italicized). Because thermal weight loss in the sauna (when necessary) always followed fluid abstinence, this interaction term can be interpreted as the added effect of dehydrating in the sauna after abstaining from fluid overnight, accounting for the individual effects of thermal exposure and fluid abstinence.

Model weights were assessed for significance using the Wald Test. Pairwise dependent 2-tailed t-tests were also used to check our hydration manipulation. Measures of central tendency are reported: mean ± standard deviation. Values greater than 3 standard deviations from the mean were removed within levels and conditions of the raw data. All models were assessed visually and revealed no violations of any statistical assumptions. Alpha was set to 0.05. All analyses were completed in the R programming language (Version 1.1.453, RStudio, Inc., Boston, USA).

## Results

### Weight loss manipulation check

All participants completed study testing. Participants completed a mean of 53.58 ± 11.79 min in the sauna during the SAU condition and 36.41 ± 21.65 min in the sauna during the FA + SAU condition. The 3 hydration conditions successfully resulted in ranging total%BMC (1.73% − -3.88%), abstinence%BMC (1.68% − -2.36%), and thermal%BMC (0.47% − -4.09%; Table 2). Positive values represent a gain in weight and negative values represent a loss of weight.

**Table 2:** Descriptive statistics for total%BMC, abstinence%BMC, and thermal%BMC resulting from the hydration manipulations

	*M*	*SD*	*Mdn*	*Min*	*Max*
Total%BMC	-0.87	1.3	-0.86	1.73	-3.88
Thermal%BMC	-0.9	0.98	-0.88	0.47	-4.09
Abstinence%BMC	0.03	0.88	0.07	1.68	-2.36
%BMC post-rehydration – Total%BMC	1.19	0.87	1.39	3.27	-0.26
%BMC post-rehydration	0.32	0.77	0.41	2.05	-1.42

n = 12. Negative values indicate greater dehydration/increasing dehydration. Some positive values are expected to result from CON test days where fluid consumption was unrestricted over the period when fluid abstinence and thermal exposure would have otherwise occurred. M = mean, SD = standard deviation, Mdn = median, Min = minimum, Max = maximum

Comparisons (pairwise dependent t-tests) of the weight changes across the 3 weight-loss conditions indicate that the total%BMC, abstinence%BMC, and thermal%BMC are related to the hydration manipulations, as expected (Table 3). SAU and FA + SAU days resulted in greater total weight loss and thermal weight loss than CON days. FA + SAU days resulted in greater fluid abstinence weight loss than SAU or CON days. Participants on average consumed 223% of their total weight loss in water (kg) during the rehydration window.

**Table 3:** Inferential comparisons of total%BMC, abstinence%BMC, and thermal%BMC between hydration manipu*lations*

	Mean difference	*t*-score
*Total weight loss (total%BMC)*		
CON	*Reference*	
SAU	-1.82	4.01**
FA + SAU	-2.15	6.11***
*Thermal weight loss (thermal%BMC)*		
CON	*Reference*	
SAU	-1.76	6.03***
FA + SAU	-1.04	4.65***
*Fluid abstinence weight loss (abstinence%BMC)*		
CON	1.11	-2.79*
SAU	1.05	-3.86**
FA + SAU	*Reference*	

n = 12. Negative values indicate decreasing hydration. T-tests are dependent and include 11 degrees of freedom. T-score = t-score derived from pairwise dependent t-tests against the “reference” category. ***p < 0.001; **p < 0.01; *p < *0.05*

### Hydration variables

Perception of thirst, symptom number, and symptom severity scores on test days were converted to Z-scores before analysis using a mean and standard deviation that was derived from questionnaires completed on their baseline weigh-in days (see Table 4). Linear mixed-effects models demonstrated that the observed changes in weight were directionally related to these measures of hydration as would be expected if these weight changes were due to dehydration (Table 5). Except for the number of symptoms experienced at the official weigh-in (LR = 6.23, p = 0.044) and their severity (LR = 6.84, p = 0.033), measures of hydration were not better explained by the separation of total%BMC into thermal%BMC and abstinence%BMC (p > 0.05).

**Table 4:** Descriptive statistics for each outcome variable

	*M*	*SD*	*Mdn*	*Min*	*Max*
*Hydration variables (At weigh-in)*					
Urine osmolality (mmol/L)	747.71	306.76	860.50	128.33	1148.00
Plasma osmolality (mmol/L)	296.30	8.56	297.00	275.33	310.67
Symptom number (Z)	1.02	1.89	0.10	-0.83	5.67
Symptom severity (Z)	2.43	3.60	0.74	-0.68	14.45
Thirst (Z)	3.13	7.05	1.26	-3.77	25.51
*Consumption (during rehydration)*					
Carbohydrates (g)	153.62	48.17	154.00	70.00	254.00
Protein (g)	18.17	7.90	18.00	7.20	38.00
Fat (g)	21.25	11.76	26.00	1.50	45.50
Water (mL)	1396.24	570.93	1438.75	295.50	2956.00
Total calories (kcal)	856.46	277.40	890.00	335.00	1300.00
*Hydration variables (post rehydration)*					
Urine osmolality (mmol/L)	648.35	350.84	748.17	66.67	1109.33
Plasma osmolality (mmol/L)	295.25	5.33	295.67	284.67	306.67
Symptom number (Z)	0.85	2.57	-0.05	-0.83	12.33
Symptom severity (Z)	2.03	4.79	0.64	-1.41	21.81
Thirst (Z)	-2.66	2.05	-2.58	-7.49	0.78
*Rowing ergometer performance variables*					
Total incremental test watts (W)	72,415.81	16,478.88	71,060.00	37,494.00	113,641.00
Average 10-stroke peak power (W)	680.32	73.03	678.75	540.50	806.00
Average 10-stroke stroke rate (strokes/min)	38.32	2.20	38.75	32.50	43.50
*Maximal energy production variables*					
Max VO_2_/kg (L/min)	54.99	5.30	55.59	44.66	67.49
Max lactate (mmol/L)	13.80	2.44	13.35	9.30	19.30
Resting blood lactate (mmol/L)	3.03	2.44	2.55	0.93	16.20
*Submaximal energy production variables*					
VO_2_ at 2 mmol/L (L/min)	37.74	6.39	38.53	28.83	50.54
VO_2_ at 4 mmol/L (L/min)	43.28	5.70	43.64	33.20	55.32
Wattage at 2 mmol (W)	185.17	45.87	183.59	95.00	284.76
Wattage at 4 mmol (W)	231.81	45.94	234.55	101.00	329.36
*Visuomotor interception task*					
Logit percent success	-1.56	1.68	-1.39	-4.60	1.39
Target-finger position difference	299.52	305.99	311.66	-222.33	1008.45
Target-finger time difference	-56.43	42.21	-59.31	-146.79	23.61
*Visuomotor stop-signal task*					
Logit successful inhibitions (%)	-0.14	2.41	-0.26	-4.60	4.60
*Visuomotor double-step task*					
Mean absolute error (BWD; mm)	2.78	0.91	2.67	1.29	5.02
Mean absolute error (FWD; mm)	2.90	1.12	3.02	1.03	5.48
Mean movement time (BWD; ms)	317.21	103.54	294.79	205.93	604.64
Mean movement time (FWD; ms)	346.64	89.81	334.46	190.92	564.98

M = mean, SD = standard deviation, Mdn = median, Min = minimum, Max = maximum, Z = Z-score units calculated using each participants’ mean and standard deviation from their baseline weigh-in days; FWD = Forward jump; BWD = Backward jump. Variables corresponding to the stop-signal task are given for the 200 ms delay condition. Variables corresponding to the interception task are given for the fast, acceleration condition

**Table 5:** Output from linear mixed effects models predicting participant thirst, symptom number, and symptom score at the official weigh-in time

Predictors	Model 1			Model 2			LRT	
	*SE*	*b (CI)*	*p*	*SE*	*b (CI)*	*p*	*LR*	*p*
Thirst (z-score)							2.65	0.266
(Intercept)	**1.52**	**1.06 (-1.99 – 4.11)**	**.493**	*1.64*	*0.46 (-2.75 – 3.67)*	*.783*		
Total	**0.73**	**-2.37 (-3.83 – -0.91)**	**.003**					
Thermal				*1.06*	*-3.12 (-5.19 – -1.05)*	*.008*		
Abstinence				*1.5*	*-0.55 (-3.49 – 2.40)*	*.720*		
Thermal*Abstinence				*1.78*	*2.75 (-0.74 – 6.25)*	*.137*		
Symptom Number (z-score)							**6.23**	**0.044**
(Intercept)	*0.49*	*0.77 (-0.20 – 1.75)*	*.125*	**0.54**	**0.63 (-0.42 – 1.68)**	**.253**		
Total	*0.17*	*-0.28 (-0.62 – 0.06)*	*.110*					
Thermal				**0.24**	**-0.41 (-0.88 – 0.05)**	**.098**		
Abstinence				**0.34**	**-0.33 (-1.00 – 0.34)**	**.342**		
Thermal*Abstinence				**0.42**	**-0.64 (-1.45 – 0.18)**	**.141**		
Symptom Severity (z-score)							**6.84**	**0.033**
(Intercept)	*0.82*	*1.94 (0.29 – 3.59)*	*.027*	**0.89**	**1.52 (-0.23 – 3.28)**	**.103**		
Total	*0.42*	*-0.56 (-1.42 – 0.29)*	*.195*					
Thermal				**0.56**	**-0.95 (-2.05 – 0.16)**	**.108**		
Abstinence				**0.8**	**-0.73 (-2.30 – 0.84)**	**.374**		
Thermal*Abstinence				**0.95**	**-1.64 (-3.51 – 0.23)**	**.100**		
Urine Osmolality (mmol/L)							1.05	0.592
(Intercept)	**58.85**	**609.15 (490.42 – 727.88)**	** < .001**	*67.67*	*612.97 (479.92 – 746.02)*	< *.001*		
Total	**30.55**	**-149.8 (-211.43 – -88.17)**	** < .001**					
Thermal				*44.56*	*-145.24 (-232.84 – -57.63)*	*.004*		
Abstinence				*64.86*	*-143.66 (-271.18 – -16.15)*	*.039*		
Thermal*Abstinence				*74.17*	*54.59 (-91.24 – 200.42)*	*.471*		
Plasma Osmolality (mmol/L)							0.46	0.795
(Intercept)	**1.52**	**293.5 (290.44 – 296.57)**	** < .001**	*1.83*	*293.41 (289.81 – 297.00)*	< *.001*		
Total	**0.97**	**-3.38 (-5.35 – -1.42)**	**.002**					
Thermal				*1.4*	*-3.53 (-6.28 – -0.77)*	*.021*		
Abstinence				*1.98*	*-2.68 (-6.58 – 1.22)*	*.193*		
Thermal*Abstinence				*2.22*	*1.4 (-2.98 – 5.77)*	*.538*		

SE = Standard error, b = Beta coefficient, CI = Confidence interval, p = p-value, LR = Likelihood ratio, LRT = Likelihood ratio test. In each case, the interpreted model (assessed by the LRT) has been bolded when the overall model fit was significant; the uninterpreted model was italicized. All models contain a random intercept for each participant. Model 1 enters the total weight loss achieved (thermal weight loss + fluid abstinence weight loss) as a predictor. Model 2 enters thermal weight loss and fluid abstinence weight loss as separate predictors. Likelihood ratio tests determine how many times better model 2 is than model 1

While no sole fixed effects were significant alone, thermal%BMC (b = -0.41, CI = [-0.88, 0.05], p = 0.098) predicted greater increases in symptom number than abstinence%BMC (b = -0.33, CI = [-1.00 – 0.34], p = 0.342). Increasing thermal%BMC also predicted greater symptom severity (b = -0.95, CI = [-2.05, 0.16], p = 0.108) than equivalently increasing abstinence%BMC (b = -0.73, CI = [-2.30, 0.84], p = 0.374).

### Visuomotor performance

#### Double-step task

The average time between target appearance and target touch (movement time) as well as the average absolute distance from the target (absolute error) was calculated. Movement time was explained 17.64 times (p = 0.001) better by the separation of total%BMC into thermal%BMC and abstinence%BMC (Table 6). While statistically accounting for the jump direction of the target and its interactions with thermal and fluid abstinence weight loss, increasing abstinence%BMC was associated with a 38.06 ms increase in movement time (b = -38.06, CI = [-62.09, 14.03], p = 0.004) for every 1% increase in %BMC. By contrast, increases in thermal%BMC (b = 30.44, CI = [13.64, 47.23], p = 0.001) and its interaction with abstinence%BMC (b = -30.49, CI = [-60.09, -0.89], p = 0.057) predicted lower movement time. These changes in movement time could not be explained by associated changes in the absolute error (p > 0.05).

**Table 6:** Output from Linear Mixed Effects Models Predicting Performance Variables

Predictors	Model 1			Model 2			LRT	
	*SE*	*b (CI)*	*p*	*SE*	*b (CI)*	*p*	*LR*	*p*
DS: mean movement time (ms)							**17.64**	**0.001**
(Intercept)	*23.27*	*334.3 (289.00 – 379.59)*	< *.001*	**21.11**	**359.01 (319.09 – 398.93)**	** < .001**		
Direction	*12.63*	*32.9 (8.32 – 57.47)*	*.012*	**13.22**	**28.29 (3.29 – 53.30)**	**.037**		
Total	*6.14*	*2.71 (-9.23 – 14.66)*	*.660*					
Direction*Total	*8.15*	*13.85 (-2.00 – 29.70)*	*.095*					
Thermal				**8.88**	**30.44 (13.64 – 47.23)**	**.001**		
Abstinence				**12.71**	**-38.06 (-62.09 – -14.03)**	**.004**		
Direction*Thermal				**10.18**	**8.38 (-10.87 – 27.63)**	**.414**		
Direction*Abstinence				**14.32**	**26.58 (-0.49 – 53.66)**	**.069**		
Thermal*Abstinence				**15.65**	**-30.49 (-60.09 – -0.89)**	**.057**		
Direction*Thermal* Abstinence				**16.2**	**14.8 (-15.83 – 45.42)**	**.365**		
DS: Mean Absolute Error (mm)							0.62	0.961
(Intercept)	0.22	2.77 (2.35 – 3.19)	< .001	*0.24*	*2.8 (2.34 – 3.25)*	< *.001*		
Direction	0.17	0.08 (-0.25 – 0.40)	.651	*0.19*	*0.13 (-0.23 – 0.50)*	*.497*		
Total	0.08	-0.08 (-0.24 – 0.08)	.326					
Direction*Total	0.11	-0.01 (-0.22 – 0.20)	.904					
Thermal				*0.13*	*-0.05 (-0.29 – 0.18)*	*.674*		
Abstinence				*0.18*	*-0.12 (-0.46 – 0.22)*	*.513*		
Direction*Thermal				*0.15*	*0.05 (-0.23 – 0.33)*	*.733*		
Direction*Abstinence				*0.21*	*-0.13 (-0.53 – 0.26)*	*.525*		
Thermal*Abstinence				*0.22*	*-0.02 (-0.43 – 0.39)*	*.919*		
Direction*Thermal* Abstinence				*0.24*	*-0.1 (-0.55 – 0.35)*	*.666*		
SS: Logit Successful Inhibitions (%)							2.07	0.355
(Intercept)	0.66	-0.09 (-1.41 – 1.24)	.899	*0.68*	*0.15 (-1.17 – 1.48)*	*.823*		
Total	0.16	0.07 (-0.25 – 0.39)	.679					
Thermal				*0.26*	*0.34 (-0.16 – 0.84)*	*.197*		
Abstinence				*0.37*	*-0.37 (-1.09 – 0.34)*	*.318*		
Thermal*Abstinence				*0.45*	*-0.44 (-1.33 – 0.45)*	*.340*		
INT: Time Difference (ms)							1.29	0.524
(Intercept)	11.38	-58.91 (-81.78 – -36.04)	< .001	*12.07*	*-60 (-83.66 – -36.34)*	< *.001*		
Total	2.99	-2.84 (-8.85 – 3.16)	.351					
Thermal				*4.76*	*-3.78 (-13.12 – 5.55)*	*.436*		
Abstinence				*6.82*	*-3.33 (-16.70 – 10.03)*	*.630*		
Thermal*Abstinence				*8.42*	*-5.5 (-22.00 – 11.00)*	*.521*		
INT: Position Difference (mm)							0.6	0.742
(Intercept)	82.64	321.7 (155.56 – 487.84)	.001	*88.35*	*319.76 (146.53 – 492.98)*	*.002*		
Total	20.69	25.42 (-16.17 – 67.01)	.232					
Thermal				*33.38*	*21.78 (-43.67 – 87.24)*	*.521*		
Abstinence				*47.8*	*40.48 (-53.25 – 134.21)*	*.407*		
Thermal*Abstinence				*59.11*	*38.09 (-77.80 – 153.99)*	*.526*		
INT: Logit Success (%)							0.34	0.841
(Intercept)	0.42	-1.51 (-2.36 – -0.66)	.002	*0.48*	*-1.46 (-2.39 – -0.53)*	*.006*		
Total	0.17	0.06 (-0.28 – 0.40)	.740					
Thermal				*0.26*	*0.12 (-0.39 – 0.63)*	*.657*		
Abstinence				*0.37*	*-0.09 (-0.82 – 0.64)*	*.815*		
Thermal*Abstinence				*0.45*	*-0.26 (-1.14 – 0.63)*	*.575*		

SE = Standard error, b = Beta coefficient, CI = Confidence interval, p = p-value, LR = Likelihood ratio, LRT = Likelihood ratio test, DS = Double-Step Task, SS = Stop-Signal Task, INT = Interception Task. In each case, the interpreted model (assessed by the LRT) has been bolded when the overall model fit was significant; the uninterpreted model was italicized. All models contain a random intercept for each participant. Model 1 enters the total weight loss achieved (thermal weight loss + fluid abstinence weight loss) as a predictor. Model 2 enters thermal weight loss and fluid abstinence weight loss as separate predictors. Likelihood ratio tests determine how many times better model 2 is than model 1

#### Stop-signal task

The logit-transformation of the percent of successful trials on the stop-signal task was determined. The proportion of successful trials was logit transformed to produce a normally distributed dependent measured that was unconstrained by 0 and 1. No weight change measure was significantly associated with this variable (p > 0.05).

#### Interception task

The absolute error in time between finger and target arrival, the absolute error in position between finger and target arrival, and the logit-transformation of the percent of successful interceptions during the interception task were calculated. No weight change measure was significantly associated with any of these variables (p > 0.05).

### Peak power test

The average of the highest wattage achieved on the two 10-stroke ergometer tests was calculated. No weight change measure, other than abstinence%BMC, was significantly associated with this variable (p > 0.05; Table 7). While greater abstinence%BMC (b = 12.14, CI = [1.96, 22.31], p = 0.029) was related to a 12.14 W reduction in performance for every %BMC achieved within the model containing abstinence%BMC and thermal%BMC, we required the model containing abstinence%BMC and thermal%BMC to better explain the data than model containing total%BMC in order to be interpreted.

**Table 7:** Output from linear mixed effects models predicting performance variables

Predictors	Model 1			Model 2			LRT	
	*SE*	*b (CI)*	*p*	*SE*	*b (CI)*	*p*	*LR*	*p*
Peak power (W)							4.8	0.091
(Intercept)	21.32	682.67 (639.82 – 725.53)	< .001	*21.28*	*677.31 (635.58 – 719.04)*	< *.001*		
Total	2.33	2.7 (-1.99 – 7.38)	.259					
Thermal				*3.62*	*-3.4 (-10.50 – 3.70)*	*.358*		
Abstinence				*5.19*	12.14 (1.96 – 22.31)*	*.029*		
Thermal*Abstinence				*6.49*	*8.59 (-4.14 – 21.32)*	*.200*		
Total Watts (W)							**9.37**	**0.009**
(Intercept)	*4720.3*	*72,882.53 (63,393.04 – 82,372.02)*	< *.001*	**4634.09**	**70,764.81 (61,678.85 – 79,850.77)**	** < .001**		
Total	*690.7*	*534.82 (-853.74 – 1923.38)*	*.447*					
Thermal				**981.14**	**-1874.35 (-3798.05 – 49.35)**	**.070**		
Abstinence				**1407.08**	**4261.51 (1502.68 – 7020.34)**	**.006**		
Thermal*Abstinence				**1756.81**	**3412.85 (-31.69 – 6857.39)**	**.066**		
Max VO_2_/kg (L/min)							1.63	0.444
(Intercept)	1.49	55.23 (52.23 – 58.24)	< .001	*1.57*	*55.01 (51.93 – 58.09)*	< *.001*		
Total	0.25	-0.28 (-0.79 – 0.23)	.273					
Thermal				*0.41*	*-0.05 (-0.85 – 0.75)*	*.906*		
Abstinence				*0.58*	*-0.5 (-1.64 – 0.65)*	*.404*		
Thermal*Abstinence				*0.73*	*-0.19 (-1.62 – 1.24)*	*.796*		
VO_2_ at 2 mmol/L (L/min)						1.19	0.551	
(Intercept)	1.85	37.82 (34.10 – 41.55)	< .001	*1.92*	*38.01 (34.25 – 41.78)*	< *.001*		
Total	0.16	0.09 (-0.23 – 0.41)	.579					
Thermal				*0.26*	*0.31 (-0.21 – 0.83)*	*.257*		
Abstinence				*0.38*	*-0.25 (-1.00 – 0.49)*	*.517*		
Thermal*Abstinence				*0.48*	*-0.32 (-1.26 – 0.61)*	*.503*		
VO_2_ at 4 mmol/L (L/min)						0.23	0.89	
(Intercept)	1.64	43.36 (40.06 – 46.66)	< .001	*1.7*	*43.46 (40.12 – 46.80)*	< *0.001*		
Total	0.19	0.09 (-0.29 – 0.48)	.627					
Thermal				*0.32*	*0.21 (-0.42 – 0.84)*	*.520*		
Abstinence				*0.46*	*-0.09 (-1.00 – 0.82)*	*.848*		
Thermal*Abstinence				*0.58*	*-0.19 (-1.32 – 0.95)*	*.752*		

SE = Standard error, b = Beta coefficient, CI = Confidence interval, p = p-value, LR = Likelihood ratio, LRT = Likelihood ratio test. In each case, the interpreted model (assessed by the LRT) has been bolded when the overall model fit was significant; the uninterpreted model was italicized. All models contain a random intercept for each participant. Model 1 enters the total weight loss achieved (thermal weight loss + fluid abstinence weight loss) as a predictor. Model 2 enters thermal weight loss and fluid abstinence weight loss as separate predictors. Likelihood ratio tests determine how many times better model 2 is than model 1

### *Incremental VO*_*2*_* max test*

#### Total test wattage

The wattage produced on each stroke of the incremental VO_2_max test was summed to determine the total test wattage produced. Total test wattage was explained 6.59 times better (p = 0.037) by the separation of weight loss into thermal%BMC and abstinence%BMC (Table 7). While thermal%BMC (b = -1874.35, CI = [-3798.05, 49.35], p = 0.070) and the interaction of thermal%BMC and abstinence%BMC (b = 3412.85, CI = -31.69, 6857.39], p = 0.066) failed to demonstrate an association with total wattage production, weight lost through fluid abstinence was associated with a 4261.51 W decrease in the total wattage produced for every 1%BMC achieved (b = 4261.51, CI = [-1502.68, -7020.34], p = 0.006).

#### ***VO***_***2***_***max***

To calculate VO_2_max per kilogram body weight (VO_2_max/kg), the highest 30 s average of oxygen consumption during each incremental VO_2_max test was retrieved and divided by the participant’s baseline weight (kg). No weight change measure was significantly associated with this variable (p > 0.05).

#### Submaximal oxygen consumption

To obtain measures of submaximal oxygen consumption, oxygen data from each stage of the incremental VO_2_max test was trimmed (to allow oxygen consumption to plateau and limit influences of participant behaviour) to include 1 min and 45 s of data occurring 2 min before the end of the stage. The trimmed oxygen data was averaged for each stage and the first stage was dropped to limit influence from the rest period. The average stroke wattage for these periods was similarly recorded. A log-linear (oxygen consumption, wattage) model was fit to the data from each test and used to determine the oxygen consumption (L/min) at the wattage that produced 2 mmol/L blood lactate and 4 mmol/L blood lactate on CON day. No weight change measure was significantly associated with either of these variables (p > 0.05).

### 2 mmol/L and 4 mmol/L blood lactate

The wattages that elicited 2 mmol/L and 4 mmol/L blood lactate was determined for each incremental VO_2_max test by fitting a cubic-linear model to the lactate readings and wattage data collected during the test and solving for these values. The 2 mmol/L (LR = 12.78, p = 0.002) and 4 mmol/L (LR = 12.78, p = 0.002) blood lactate variables were both better fit by the model that included thermal%BMC and abstinence%BMC (Table 8). In the 2 mmol/L (b = 27.84, CI = [14.69, 40.99], p < 0.001) and 4 mmol/L (b = 20.45, CI = [8.91, 31.99], p = 0.002) models, each 1%BMC achieved through fluid abstinence predicted a reduction of 27.84 W and 20.45 W in the wattage required to elicit these blood lactate concentrations, respectively. By contrast, weight lost thermally did not have a meaningful effect on the wattages required to elicit concentrations of 2 mmol/L (b = -9.50, CI = [-18.68, 0.31], p = 0.055) and 4 mmol/L (b = 0.22, CI = [-4.31, 4.74], p = 0.926) blood lactate. The effect of preceding thermal weight loss with fluid abstinence had inconsistent effects on 2 mmol/L (b = 22.46, CI = [6.18, 38.75], p = 0.013) and 4 mmol/L (b = -0.97, CI = [-9.02, 7.07], p = 0.815) blood lactate. No weight changes measures were meaningfully related to resting or maximal blood lactate values (p > 0.05), suggesting that the observed faster rate of lactate accumulation is not explained by a higher lactate at the start of exercise or a lower achievable lactate concentration.

**Table 8:** Output from linear mixed effects models predicting performance variables

Predictors	Model 1			Model 2			LRT	
	*SE*	*b (CI)*	*p*	*SE*	*b (CI)*	*p*	*LR*	*p*
Resting Blood Lactate (mmol/L)							0.81	0.67
(Intercept)	0.5	2.9 (1.90 – 3.90)	< .001	*0.58*	*2.74 (1.61 – 3.86)*	< *.001*		
Total	0.32	-0.16 (-0.80 – 0.49)	.630					
Thermal				*0.44*	*-0.32 (-1.18 – 0.55)*	*.483*		
Abstinence				*0.62*	*-0.09 (-1.31 – 1.13)*	*.886*		
Thermal*Abstinence				*0.7*	*-0.33 (-1.71 – 1.05)*	*.641*		
Wattage at 2 mmol (W)							**12.78**	**0.002**
(Intercept)	*12.1*	*189.45 (165.21 – 213.69)*	< *.001*	**13.01**	**176.86 (151.36 – 202.37)**	** < .001**		
Total	*3.76*	*4.91 (-2.65 – 12.47)*	*.205*					
Thermal				**4.68**	**-9.5 (-18.68 – -0.31)**	**.055**		
Abstinence				**6.71**	**27.84 (14.69 – 40.99)**	** < .001**		
Thermal*Abstinence				**8.31**	**22.46 (6.18 – 38.75)**	**.013**		
Wattage at 4 mmol (W)							**10.96**	**0.004**
(Intercept)	*12.4*	*236.91 (212.01 – 261.80)*	< *.001*	**12.84**	**227.35 (202.16 – 252.53)**	** < .001**		
Total	*3.1*	*5.84 (-0.39 – 12.07)*	*.072*					
Thermal				**4.11**	**-4.77 (-12.82 – 3.28)**	**.259**		
Abstinence				**5.89**	**20.45 (8.91 – 31.99)**	**.002**		
Thermal*Abstinence				**7.31**	**8.86 (-5.47 – 23.19)**	**.239**		
Max Lactate (mmol/L)							2.51	0.286
(Intercept)	0.58	13.6 (12.44 – 14.77)	< .001	*0.65*	*13.22 (11.96 – 14.49)*	< *.001*		
Total	0.27	-0.22 (-0.76 – 0.31)	.412					
Thermal				*0.39*	*-0.64 (-1.40 – 0.11)*	*.110*		
Abstinence				*0.55*	*0.43 (-0.65 – 1.51)*	*.440*		
Thermal*Abstinence				*0.66*	*0.41 (-0.88 – 1.70)*	*.541*		

SE = Standard error, b = Beta coefficient, CI = Confidence interval, p = p-value, LR = Likelihood ratio, LRT = Likelihood ratio test. In each case, the interpreted model (assessed by the LRT) has been bolded when the overall model fit was significant; the uninterpreted model was italicized. All models contain a random intercept for each participant. Model 1 enters the total weight loss achieved (thermal weight loss + fluid abstinence weight loss) as a predictor. Model 2 enters thermal weight loss and fluid abstinence weight loss as separate predictors. Likelihood ratio tests determine how many times better model 2 is than model 1

### Ruling out alternative explanations

#### Order effects

The effect of testing order (i.e. familiarity and learning) were ruled out by repeating each significant model in analysis and including testing order as a fixed effect. No significant effects of order or meaningful changes in the model weights retrieved were discovered (p > 0.05).

#### Foods and fluids over 48 h prior and during the dehydration window

To determine whether the amount of fluids (mL), carbohydrates (g), fats (g), proteins (g), sodium (mg) and total calories (kcal) consumed over 48-h prior to each testing day could explain the performance changes observed, linear mixed-effect models were created for each measure of dietary intake. No relationship between total%BMC, thermal%BMC, abstinence%BMC, or the interaction of thermal%BMC and abstinence%BMC, and any of the food and fluid variables was observed (p > 0.05).

Similar models were used to assess food and fluid consumed during the rehydration window. Greater total%BMC predicted greater consumption of carbohydrates (b = -8.66, CI = [-14.04, -3.28], p = 0.004) and fluids (b = -238.04, CI = [-359.70, -116.39], p < 0.001). Splitting total%BMC into thermal%BMC and abstinence%BMC did not improve the fit of either the model (p > 0.05). Protein consumption was 6.07 times (p = 0.048) better explained by thermal%BMC and abstinence%BMC than total%BMC alone. Greater thermal%BMC was associated with less protein consumption (b = 2.37, CI = [0.18, 4.55], p = 0.046) while greater abstinence%BMC was unrelated to protein consumption, b = -1.29, CI = [-4.41, 1.83], p = 0.427. No interactive affect was discovered, b = 0.00, CI = [-3.79, 3.80], p = 0.999. The total number of calories and amount of fat consumed was unrelated to any marker of dehydration (p > 0.05). Differences in protein consumption following thermal%BMC and abstinence%BMC may or may not contribute to our findings. However, this seems unlikely as it would suggest that less protein intake during the rehydration window was protective against weight loss.

## Discussion

Understanding the effect of weight-reducing dehydration practices on rowing performance is important to lightweight rowing coaches and athletes. We investigated the combined and individual effects of mild weight loss via fluid abstinence and thermal exposure on proxies of bodily systems that contribute to rowing performance: the aerobic system, the anaerobic lactic system, the anaerobic alactic system, and the neuromotor control system. Manipulation checks suggest that the employed weight-reducing techniques successfully resulted in weight loss that can be attributed to dehydration and can be partitioned by the protocol used to achieve it. Our primary finding was that in every case that increasing total weight loss was related to a measured proxy, the proxy was better explained by the separation of weight loss into the amount achieved through fluid abstinence and thermal exposure (i.e. dehydration technique matters). Our secondary findings were that weight lost through fluid abstinence but not thermal exposure, was associated with: (1) lower total wattage produced on the incremental VO_2_max test, (2) lower wattage at 2 mmol/L and 4 mmol/L blood lactate, and (3) slower movement time on the double-step task. In sum, our findings suggest that some bodily systems involved in rowing performance are vulnerable to weight-reducing dehydration practices within the weight loss range studied. This vulnerability seems to depend on the weight loss protocol used with fluid abstinence, but not thermal exposure, producing performance effects.

The present study supports existing evidence that weight-reducing practices can reduce aspects of rowing performance. To date, 6 randomized controlled trials have tested the effects of weight-reducing dehydration practices on rowing [3, 4, 14–17]. All used a within-subjects design, an enveloping marker of rowing performance (2000 m time trial performance) and allowed a 2-h rehydration window following dehydration. Four of these studies, which did not control the technique used to elicit weight loss, generally suggest rowing performance is reduced (~ 2 s slower) when body mass is decreased by 4–6% [3, 4, 14, 15]. However, 1 study which required athletes to engage in a period of fluid abstinence found much larger effects on performance (22 s slower) [16]. The final study utilized a similar weight loss protocol but failed to adjust for within-subjects’ dependencies in their analysis, making comparisons challenging [17]. All studies represent large effects relative to the estimated smallest meaningful change in on-water rowing performance (1.0–1.5 s for a 2000 m race) [35].

We found that fluid abstinence was more consistently associated with performance decrements than thermal exposure. No other studies have examined the effect of differing weight-reducing dehydration techniques in the rowing setting. However, a few studies have investigated this in other contexts and support an effect of dehydration protocol on performance. Caldwell et al. (1984) found that weight loss using diuretics and sauna exposure reduced performance more than exercise-induced weight loss on VO_2_max, work capacity, and measures of submaximal lactate at 4.1% BMC in 62 athletes [23]. Sauna weight loss (3.4% BMC) has also been associated with worse performance relative to diuretics (3.8% BMC) on measures of leg strength and leg force in track and field athletes and volleyball players [22]. Additionally, 2 meta-analyses have found weight-reducing dehydration practices that involve exercise and/or fluid abstinence result in poorer muscle endurance [9], lactic and alactic anaerobic capacity [9], and cognitive performance [36] than passive techniques such as sauna exposure. These studies identify weight-loss protocol as a moderator of the relationship between weight loss and performance but are inconsistent in which weight loss protocol is reported as the least harmful. Future research should investigate if the type of performance task or the provision of the rest/rehydration window prior performance influences which weight loss protocol is superior.

We discovered that weight loss through fluid abstinence was related to poorer performance on incremental VO_2_max test, but not the peak power ergometer test. Interestingly, weight loss through thermal exposure was more strongly positively related to the number and severity of dehydration symptoms than weight loss through fluid abstinence. We used the peak power ergometer test as a proxy of the anaerobic alactic energy system and did not find reductions in performance with weight loss. This finding is in agreement with other studies of moderate (2–3%) weight loss via dehydration which also found preserved peak power performance [37, 38]. Notably, greater amounts of weight loss (3–4% BMC) do seem to be able to produce poorer power performance [39]. Thus, it may be that while power and strength performance can be reduced by weight-reducing dehydration practices, our investigation did not employ a large enough magnitude of dehydration to observe performance effects.

Performance on the incremental VO_2_max test is more apt to test the lactic anaerobic and aerobic energy systems given its longer duration (~ 20 min). The identified relationship between increasing weight loss via fluid abstinence and lower total wattage production on this test may be due to changes in 1 or both energy systems. However, we report no effect of any of our weight loss measures on maximal aerobic capacity, making the aerobic energy system an unlikely candidate for this decline in performance. Instead, it may be that changes to the lactic anaerobic system explain changes in performance.

We found an earlier onset of lactate accumulation with increasing weight loss through fluid abstinence. Previous investigations in other athlete populations have found higher blood lactate at submaximal intensities with weight-reducing dehydration practices [40, 41]. Nine recreationally fit males demonstrated elevated blood lactate at all time points during a constant-load cycle-ergometer task following a 2%BMC via a warm water bath the previous evening [40]. Similarly, a study of intercollegiate wrestlers has demonstrated a lower treadmill velocity required to achieve the lactate threshold following exercise in a rubberized sweat suit [41]. Weight-reducing dehydration practices (4–6%BMC) have further been demonstrated to reduce muscular endurance performance (e.g. 30 s horizontal isometric rowing, 6-min arm crank ergometer), which should act as proxies of the anaerobic lactic system, even when a rehydration window between weight loss and performance is provided [41, 42]. Earlier lactate accumulation following weight-reducing dehydration practices exercise has been explained by increased muscle temperature and resultant greater carbohydrate oxidation [43].

In addition, we examined how neuromotor control (assessed by the visuomotor tests) and stroke efficiency (assessed by submaximal VO_2_) may be affected by weight-reducing dehydration practices. We found evidence for a reduction in participants’ capacity to alter an on-going movement (double-step task), but not their capacity to inhibit planned movements (stop-signal task) or coordinate motor timing with a moving target (interception task) when assessed part way through the rehydration window. Further, we did not find a relationship between weight loss and stroke efficiency during incremental VO_2_max test. Our findings suggest that some elements of neuromotor control may be affected by mild weight-reducing dehydration practices and that these effects depend on technique used to elicit weight loss. How/if these changes in neuromotor control impact rowing performance either on the rowing ergometer or on-water requires further investigation.

Previous research has demonstrated an effect of fluid abstinence on various tests that rely heavily on neuromotor control [44, 45]. Only 1 investigation has examined an effect of weight-reducing dehydration practices (4–6% BMC) on neuromotor control when a rehydration period was allowed. They demonstrated poorer reaction and movement time during wrestling matches despite 12 h to rehydrate and refeed before performing [46]. By contrast, weight-reducing dehydration practices that relied on sauna exposure have less consistently demonstrated an effect on neuromotor control. After eliciting 2.8% BMC in via sauna exposure in 2 separate experiments, Cian, et al. (2000) and Cian, et al. (2001) were unable to detect a change reaction time task performance in either experiment or a change in performance on a joystick tracking test in the second experiment [47, 48]. In the first experiment, sauna weight loss degraded joystick tracking performance before but not after 2 h of rehydration [47]. More work is needed to learn how potential changes in neuromotor control with weight-reducing dehydration practices might influence stroke performance mechanics and race outcomes.

### Strengths

Using experienced rowers and protocols that mimic international competition, we demonstrate relationships between weight-reducing dehydration practices and proxies of a wide number of bodily systems involved in rowing. Our statistical approach (to use %BMC as the unit of analysis) allowed us to report our findings as a change in a performance measure per %BMC which may prove valuable to some stakeholders. Further, this approach should improve statistical power of our analysis if (1) the effect of weight-reducing dehydration practices relies on the amount of weight lost through the protocol as opposed to exposure to the protocol itself, and (2) weight loss is related to performance in a dose–response relationship that can be approximated linearly. Previous work supports these assertions [8, 12, 36].

### Limitations

In the current study, rowers were unblinded to the weight manipulation which may have affected their performance. However, a lack of blinding is less likely to have affected this study given participants’ perceived dehydration symptomology was inconsistent with the effects observed. Further, all participants were high-level athletes and were aware that some data related to performance would be provided to them following all testing to be used for monitoring training, motivating them to perform. Our experimental design did not allow us to randomly assign the observed changes in body mass (the unit of analysis). As such, our study is quasi-experimental in nature and can only suggest causal relationships. Our random assignment of participants to counterbalanced testing order, careful control of the experiment environment, and assessment of potential sources of uncontrolled variance (e.g. establishing a relationship between %BMC and other markers of dehydration, diet logs) improve the likelihood that our demonstrated relationships are causal. Because the present study allowed rowers to rehydrate ad libitum, we cannot be certain that all rowers in the present study rehydrated optimally. While this may have introduced error into our findings, we chose this approach because 1) this mimics the applied setting where fluid and food consumption is unprescribed; 2) we expected rowers to attempt to rehydrate optimally because they were aware that they had experienced dehydration, were provided fluids demonstrated to maximize rehydration [15], and were advised to rehydrate aggressively; 3) this allowed a consistent experience of satiety prior testing and minimized gastric upset resulting from over or under feeding; and 4) it would have been impossible to maintain our design and prescribe fluids/foods directly in accordance to weight changes as baseline body weight measurements were recorded before, during, and after the testing period. Despite these limitations, our study provides a strong investigation of weight-reducing dehydration practices in rowers.

## Conclusions

We sought to determine whether mild weight-reducing dehydration practices compromise rowing performance. The total weight loss achieved was related to some aspects of rowing performance. When split by the manner in which it was achieved, the weight loss due to fluid abstinence, but not thermal exposure, was related to a lower total wattage produced on the incremental VO_2_max test, lower wattages required to elicit 2 mmol/L and 4 mmol/L blood lactate, and slower movement time on a visuomotor task. It may be that reductions in these bodily systems contribute to poorer rowing performance following weight-reducing dehydration practices. Overall, our findings suggest that proxies of bodily systems involved in rowing performance may be negatively affected by mild weight loss practices and that the technique by which weight loss is achieved is important.

## Supplementary Information

**Additional file 1.** Equations for partitioning percent body mass change (%BMC).

## Data Availability

The datasets used and/or analysed during the current study are available from the corresponding author on reasonable request.

## Abbreviations

VO_2_max: Maximum oxygen uptake test
%BMC: Percent body mass change
95%CI: 95% Confidence interval
SAU: Thermal exposure in sauna
FA + SAU: Fluid abstinence plus thermal exposure in sauna
CON: No weight-loss condition
total%BMC: Total change in weight
thermal%BMC: Change in weight due to thermal exposure in sauna
abstinence%BMC: Change in weight due to fluid abstinence
VAST: Visual analogue scale of thirst
SES of SCAT3: Symptom Evaluation Subscale of the Sport Concussion Assessment Test (3rd Edition)
ASA24-Canada: Automated Self-Administered 24-Hour-Canada
1%BMC: Change in the outcome measure for a 1% change in BMC
LR: Likelihood Ratio
SE: Standard error
b: Beta coefficient
CI: Confidence interval
p: P-value
LRT: Likelihood ratio test

## References

[1] Slater G, Rice A, Jenkins D, Hahn A (2014). Body mass management of lightweight rowers: nutritional strategies and performance implications. Br J Sports Med.

[2] Barrett RS, Manning JM (2004). Rowing: relationships between rigging set-up, anthropometry, physical capacity, rowing kinematics and rowing performance. Sports Biomech.

[3] Slater GJ, Rice AJ, Sharpe K, Mujika I, Jenkins D, Hahn AG (2005). Body-mass management of Australian lightweight rowers prior to and during competition. Med Sci Sports Exerc.

[4] Slater GJ, Rice AJ, Sharpe K (2005). Impact of acute weight loss and/or thermal stress on rowing ergometer performance. Med Sci Sports Exerc.

[5] de Campos MF, de Moraes Bertuzzi RC, Grangeiro PM, Franchini E (2009). Energy systems contributions in 2,000 m race simulation: a comparison among rowing ergometers and water. Eur J Appl Physiol.

[6] Cuijpers LS, Passos PJ, Murgia A, Hoogerheide A, Lemmink KA, de Poel HJ (2017). Rocking the boat: does perfect rowing crew synchronization reduce detrimental boat movements?. Scand J Med Sci Sports.

[7] Warmenhoven J, Cobley S, Draper C, Harrison AJ, Bargary N, Smith R (2017). Assessment of propulsive pin force and oar angle time-series using functional data analysis in on-water rowing. Scand J Med Sci Sports.

[8] Cheuvront SN, Kenefick RW (2014). Dehydration: physiology, assessment, and performance effects. Compr Physiol.

[9] Savoie FA, Kenefick RW, Ely BR, Cheuvront SN, Goulet ED (2015). Effect of hypohydration on muscle endurance, strength, anaerobic power and capacity and vertical jumping ability: a meta-analysis. Sports Med.

[10] Barr SI (1999). Effects of dehydration on exercise performance. Can J Appl Physiol.

[11] Prendergast HM, Bannen T, Erickson TB, Honore KR (2003). The toxic torch of the modern Olympic Games. Vet Hum Toxicol.

[12] Armstrong LE, Costill DL, Fink WJ (1985). Influence of diuretic-induced dehydration on competitive running performance. Med Sci Sports Exerc.

[13] Derave W, Clercq DD, Bouckaert J, Pannier J-L (1998). The influence of exercise and dehydration on postural stability. Ergonomics.

[14] Slater GJ, Rice AJ, Tanner R, Sharpe K, Jenkins D, Hahn AG (2006). Impact of two different body mass management strategies on repeat rowing performance. Med Sci Sports Exerc.

[15] Slater GJ, Rice AJ, Sharpe K, Jenkins D, Hahn AG (2007). Influence of nutrient intake after weigh-in on lightweight rowing performance. Med Sci Sports Exerc.

[16] Burge CM, Carey MF, Payne WR (1993). Rowing performance, fluid balance, and metabolic function following dehydration and rehydration. Med Sci Sports Exerc.

[17] Penkman MA, Field CJ, Sellar CM, Harber VJ, Bell GJ (2008). Effect of hydration status on high-intensity rowing performance and immune function. Int J Sport Physiol.

[18] Kelly, D.J., Nepotiuk, A. & Brown, L. E. (2021). Rowing performance after dehydration: an unexpected effect of method. *SportRχiv*. 10.31236/osf.io/pv9dw

[19] Tomazo-Ravnik T, Jakopič V (2006). Changes in total body water and body fat in young women in the course of menstrual cycle. Int J Anthropol.

[20] Smith MF, Newell AJ, Baker MR (2012). Effect of acute mild dehydration on cognitive-motor performance in golf. J Strength Cond Res.

[21] Aldridge G, Baker J, Davies B. Effects of hydration status on aerobic performance for a group of male university rugby players. *J Exerc Physiol Online.* 2005;8(5).

[22] Viitasalo J, Kyröläinen H, Bosco C, Alen M (1987). Effects of rapid weight reduction on force production and vertical jumping height. Int J Sports Med.

[23] Caldwell JE, Ahonen E, Nousiainen U (1984). Differential effects of sauna-, diuretic-, and exercise-induced hypohydration. J Appl Physiol.

[24] WHO. WHO guidelines on drawing blood: best practices in phlebotomy. *World Health Organization.* 2010. http://apps.who.int/iris/bitstream/handle/10665/44294/9789241599221_eng.pdf;jsessionid=2ABCDB0DE0297EADE2CC42304A0ED367?sequence=1. 23741774

[25] Rolls BJ, Wood RJ, Rolls ET, Lind H, Lind W, Ledingham JG. Thirst following water deprivation in humans. *Am J Physiol Regul Integr Comp Physiol.* 1980;239(5):476–482. 10.1152/ajpregu.1980.239.5.R476 7001928

[26] SCAT3. SCAT3. *Br J Sports Med*. 2013;47(5):259–259. 23479480

[27] Iida S. The Effects of Hypohydration on Neurocognitive, Balance, Vestibular Ocular Motor Functions and Mood State. *Theses and Dissertations.* 2016:1555. Retrieved from https://scholarworks.uark.edu/etd/1555.

[28] Bedore CD, Livermore J, Lehmann H, Brown LE (2018). Comparing three portable, tablet-based visuomotor tasks to laboratory versions: an assessment of test validity. J Concussion.

[29] Brainard DH (1997). The psychophysics toolbox. Spat Vis.

[30] Pelli D (1997). The VideoToolbox software for visual psychophysics: transforming numbers into movies. Spat Vis.

[31] Kleiner M BD, Pelli D. “What’s new in Psychtoolbox-3?” Perception 36 ECVP Abstract Supplement. 2007.

[32] Lawton TW, Cronin JB, McGuigan MR (2013). Strength, power, and muscular endurance exercise and elite rowing ergometer performance. J Strength Cond Res.

[33] Ingham S, Whyte G, Jones K, Nevill A (2002). Determinants of 2,000 m rowing ergometer performance in elite rowers. Eur J Appl Physiol.

[34] Bourdon PC, Woolford SM, Buckley JD (2017). Effects of varying the step duration on the determination of lactate thresholds in elite rowers. Int J Sport Physiol.

[35] Smith TB, Hopkins WG (2011). Variability and predictability of finals times of elite rowers. Med Sci Sports Exerc.

[36] Wittbrodt MT, Millard-Stafford M (2018). Dehydration impairs cognitive performance: a meta-analysis. Med Sci Sports Exerc.

[37] Cheuvront SN, Carter R, Haymes EM, Sawka MN (2006). No effect of moderate hypohydration or hyperthermia on anaerobic exercise performance. Med Sci Sports Exerc.

[38] Evetovich TK, Boyd JC, Drake SM (2002). Effect of moderate dehydration on torque, electromyography, and mechanomyography. Muscle Nerve.

[39] Judelson DA, Maresh CM, Anderson JM (2007). Hydration and muscular performance. Sports Med.

[40] Green JM, Miller B, Simpson J (2018). Effects of 2% dehydration on lactate concentration during constant-load cycling. J Strength Cond Res.

[41] Webster S, Rutt R, Weltman A (1990). Physiological effects of a weight loss regimen practiced by college wrestlers. Med Sci Sports Exerc.

[42] Hickner R, Horswill C, Welker J, Scott J, Roemmich J, Costill D (1991). Test development for the study of physical performance in wrestlers following weight loss. Int J Sports Med.

[43] Logan-Sprenger HM, Heigenhauser GJF, Jones GL, Spriet LL. The effect of dehydration on muscle metabolism and time trial performance during prolonged cycling in males. *Physiol Rep.* 2015;3(8):e12483. 10.14814/phy2.12483 PMC4562569 26296770

[44] Lindseth PD, Lindseth GN, Petros TV, Jensen WC, Caspers J (2013). Effects of hydration on cognitive function of pilots. Mil Med.

[45] Watson P, Whale A, Mears SA, Reyner LA, Maughan RJ (2015). Mild hypohydration increases the frequency of driver errors during a prolonged, monotonous driving task. Physiol Behav.

[46] Kraemer WJ, Fry AC, Rubin MR (2001). Physiological and performance responses to tournament wrestling. Med Sci Sports Exerc.

[47] Cian C, Barraud P, Melin B, Raphel C (2001). Effects of fluid ingestion on cognitive function after heat stress or exercise-induced dehydration. Int J Psychophysiol.

[48] Cian C, Koulmann N, Barraud PA, Raphel C, Jimenez C, Melin B (2000). Influences of variations in body hydration on cognitive function: Effect of hyperhydration, heat stress, and exercise-induced dehydration. J Psychophysiol.

