# Supplementary material for: A quasi-experimental examination of weight-reducing dehydration practices in collegiate male rowers
Source: BMC Sports Sci Med Rehabil. 2021 Sep 25;13:115. doi: 10.1186/s13102-021-00344-7 (PMC8466389; doi:10.1186/s13102-021-00344-7)
Supplement: Supplementary file 1 — Additional file 1. Equations for partitioning percent body mass change (%BMC). [file 13102_2021_344_MOESM1_ESM.docx]

**Appendix 1**

*Total%BMC = [body mass at the official weigh-in – average body mass] / [average body mass])*

Equation 1. Calculating total body mass change.

*Thermal%BMC = [body mass at the official weigh-in – body mass at arrival to the testing center] / [average body mass])*

Equation 2. Calculating body mass change during the thermal exposure period.

*Abstinence%BMC = [body mass at arrival to the testing center – average body mass] / [average body mass])*

Equation 3. Calculating body mass change during the period of fluid abstinence.

*Rehydration %BMC = ([body mass at the end of the rehydration window – body mass at the official weigh-in] / [average body mass])*

Equation 4. Calculating body mass change during the rehydration window.
